# A Two-Echelon Cooperated Routing Problem for a Ground Vehicle and Its Carried Unmanned Aerial Vehicle

**DOI:** 10.3390/s17051144

**Published:** 2017-05-17

**Authors:** Zhihao Luo, Zhong Liu, Jianmai Shi

**Affiliations:** Science and Technology on Information System Engineering Laboratory, National University of Defense Technology, Changsha 410073, China; liuzhong@nudt.edu.cn (Z.L.); jianmaishi@nudt.edu.cn (J.S.)

**Keywords:** vehicle routing, two-echelon routing, vehicle-carried UAVs, unmanned aerial vehicle routing

## Abstract

In this paper, a two-echelon cooperated routing problem for the ground vehicle (GV) and its carried unmanned aerial vehicle (UAV) is investigated, where the GV travels on the road network and its UAV travels in areas beyond the road to visit a number of targets unreached by the GV. In contrast to the classical two-echelon routing problem, the UAV has to launch and land on the GV frequently to change or charge its battery while the GV is moving on the road network. A new 0–1 integer programming model is developed to formulate the problem, where the constraints on the spatial and temporal cooperation of GV and UAV routes are included. Two heuristics are proposed to solve the model: the first heuristic (H1) constructs a complete tour for all targets and splits it by GV routes, while the second heuristic (H2) constructs the GV tour and assigns UAV flights to it. Random instances with six different sizes (25–200 targets, 12–80 rendezvous nodes) are used to test the algorithms. Computational results show that H1 performs slightly better than H2, while H2 uses less time and is more stable.

## 1. Introduction

Due to the rapid development in embedded control systems, miniaturization, sensors, and communication technologies in recent decades, a variety of small and low cost unmanned aerial vehicles (UAVs) are developed as remote sensors, which have been widely used in military and civil areas [1]. When a traditional manned ground vehicle is armed with a UAV and works cooperatively, it is thought to be able to complete some tasks more efficiently with lower cost, such as goods delivery [2], mapping [3], bound patrolling [4], surveillance applications, and sensor data gathering [5] together with search and rescue missions. Amazon developed a fleet of UAVs for small parcel delivery [6]. Based on the application background of Amazon, Murray and Chu [2] investigated the UAV-assisted parcel delivery problem and shows that GV cooperating with a UAV can greatly increase the efficiency of the “last-mile’’ parcel delivery. As its extension, UPS started its UAVs delivery test in February 2017. The drone flied up to the drop off location, released the package and autonomously returned to the vehicle. However, the GV do not have to wait for the drone to return, they can just head off towards the next delivery. In the 2016 International Consumer Electronics Show (CES), two famous corporations DJI-Innovations and Ford proposed a novel mode for wild search, where the F-150 carried a UAV and conducted the search for multiple targets in the wilderness cooperatively [7]. Coincidentally, a new concept vehicle with UAV was also developed by Rinspeed at CES 2016, where the UAV provided a monitoring service to expand the vision for real-time vehicle control [8]. According to Rinspeed, the UAV should be able to take off from the moving vehicle to complete some mission and return to the vehicle automatically.

**Figure 1:**
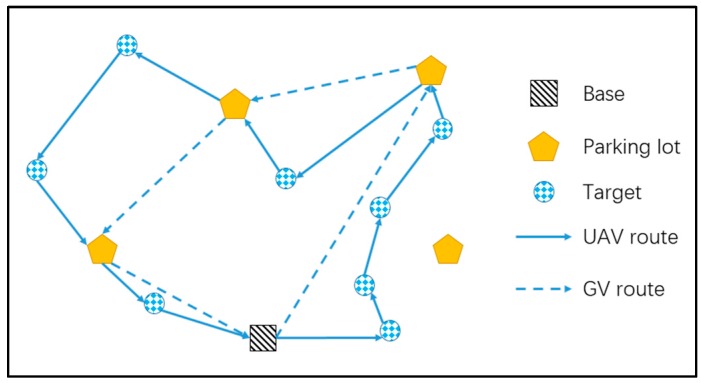
Schematic diagram of the two-echelon GV and UAV cooperated routing problem (2E-GU-RP).

When the GV and UAV cooperatively work for delivery, rescue, or patrolling, a variety of new challenges are generated in their routing problem, which has recently also attracted increasing attention from many researchers [2,9]. In this paper, we study a two-echelon GV and UAV cooperated routing problem (2E-GU-RP) which proposes a new routing strategy, where the GV travels on the road network and its UAV travels in areas beyond the road for visiting GV-unreachable targets. As shown in Figure 1, the GV carries the UAV, starts from the base, takes a round trip on the road network and then travels back to the base. There are a number of rendezvous points (parking lots) in the road network, at which they are available for the GV to stop and allow the UAV to take off or/and land. There are a set of predetermined targets located outside the road, which can only be visited by the UAV. The mission is to minimize the time of the UAV route with its endurance concerned. Thus, there are three critical decisions to be made: planning the GV’s route, determining where to stop the GV, and planning the UAV’s route for visiting targets during each flight. 

To solve the above GV and UAV routing problem, we develop a 0–1 integer programming model to formulate the problem. Two heuristics are designed to search better feasible solutions for the problem, which are tested by randomly generated instances. An exhaustive method is also developed for obtaining exact optimal solutions of small-size instances, and compared with the two heuristics.

The remainder of this paper is arranged as follows: The existing literature is described in Section 2. In Section 3, we elaborate our problem with a mathematical model. Section 4 details the proposed heuristic algorithms. In Section 5, we discuss the computational study. Finally, conclusions are drawn and directions in future research are highlighted in Section 6.

## 2. Literature Review

In the proposed 2E-GU-RP problem, we aim at optimizing the routes for both the GV and UAV, and the two most relevant streams of literature are UAV routing problem and two-echelon location routing problem, which are reviewed as follows.

In recent years, UAVs have been widely used in military and civil operations, and their flight routing problem has also become an important research topic in the area of operations research. Shetty [4] considered a strategic routing of a fleet of UAVs to finish surveillance mission. Mufalli [10] constructed a new mathematical model for UAV routing to collect data from simultaneous sensors. Similar research on data collection was investigated by Ozbaygin [11], which was studied as a time-constrained maximal covering salesman problem. Avellar [12] developed a group of UAVs for area coverage and Vanegas [13] researched the routing problem with environmental uncertainty. Additionally, Sabo devoted frameworks of UAV routing with communication considerations [14], and Rasmussen [15] proposed a cooperative multi-UAVs path planning method.

**Figure 2:**
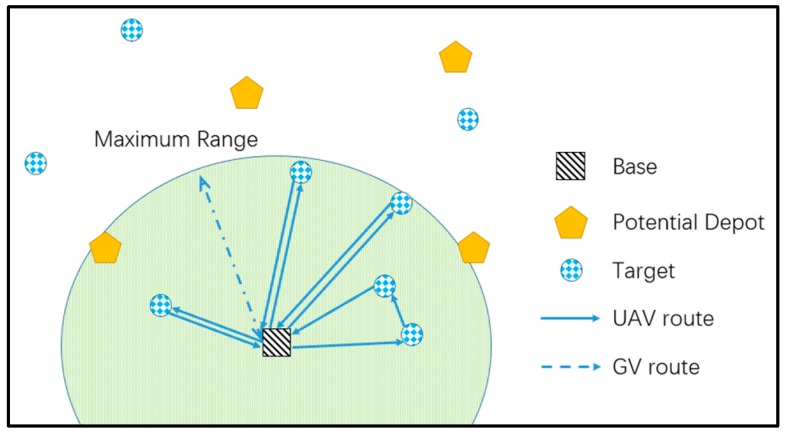
Schematic diagram of the unmanned aerial vehicle (UAV) Routing Problem.

Figure 2 illustrates a typical UAV routing problem. Due to the restrictions on endurance and communication distance, each UAV starts from the base to visit several targets and returns to the base, while some targets out of the communication and endurance ranges cannot be visited. In the UAV routing problem, the base is a fixed facility which limits the visiting range of the UAV, while in our problem the GV acts as a moving base and extends the area served by the UAV. 

**Figure 3:**
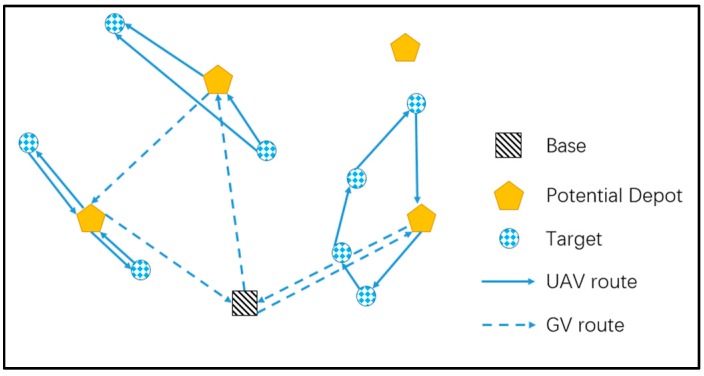
Schematic diagram of the two-echelon location and routing problem (2E-LRP).

The two-echelon location and routing problem (2E-LRP) is modeled to improve the efficiency of goods distribution [16], whose schematic diagram is shown in Figure 3. The 2E-LRP includes two types of fundamental problems, in which a manager should determine depot locations and determine the first level route for distribution of products to open depot together with the second level routes for delivery of goods to customers [17]. Most of the 2E-LRPs employ larger vehicles to distribute goods to open depots and smaller vehicles to deliver goods to customers [18]. Manyam [19] investigated a special cooperative routing problem where there was a larger vehicle acting as a temporary depot and equipped with one UAV to complete some intelligence, surveillance, or reconnaissance (ISR) tasks. The research of cooperative routing strategies in [19] can be categorized by synchronization constraints, which were discussed by Drexl [20]. With no synchronization concerned, we could regard the cooperative routing problem as an extension of the conventional two-echelon routing problem by routing the ground vehicle in the first echelon and routing the UAV in the second echelon. However, in their problem, the vehicle has to stay still during the flight of the UAV. In our problem, the GV keeps on moving during the routing of UAV, which can significantly improve the efficiency and flexibility of conducting ISR tasks by UAV.

2E-LRP has some similar modeling characteristics to our problem, such as the restriction on the endurance of the second level vehicles, the selection of locations from a set of potential locations, and the planning progress of routes for two echelons. Despite the mentioned similarities, there are several critical differences between 2E-LRP and our problem, which can be summarized as follows:

*Spatial cooperation* in traditional 2E-LRP: the spatial routes in two echelons are independent of each other, so a change for a route in the second echelon usually does not affect the routes in the first echelon. However, in our problem, when the GV is able to travel along a certain road arc, the UAV must visit the targets around the same arc at the same time due to the restriction of the UAV’s endurance. Thus, when either the GV or UAV changes its route, the other one usually has to change accordingly.

*Temporal cooperation* in traditional 2E-LRP: the routing periods for routes in two echelons are independent of each other, and usually some larger vehicles are employed in the first echelon to distribute large amounts of products to open depot in a low-period cycle (e.g., per week or per month), while smaller vehicles are employed in the second echelon to deliver products to customers in a high-period cycle (e.g., per day or per week). In our problem, both the GV and UAV must travel in their routes synchronously. Each road arc traversed by the GV corresponds to a flight route of the UAV, and both of them are restricted by the endurance of the UAV. Further, both the GV and UAV have to leave the head of the road arc at the same time, reach the tail of the road arc, wait for each other in order to change or charge the UAV’s battery, and start the following mission.

With the development of remote control technology and autonomous control technology, cooperative routing problems draw a significant amount of interest from academia, industry, and government agencies [21]. Passino [22] applied the cooperative task scheduling method to autonomously networked ground vehicles. One broad categorization of related problems involves surveillance applications with cooperative air and ground assets [5,23]. There are also certain kinds of cooperative routing problems for GV and UAV, called TSP with drone (TSP-D) [24,25,26,27] and VRP with drone (VRP-D) [28,29]. In these problems, the UAV is carried by a mobile platform that keeps on moving on its own route while UAV executes its mission. Furthermore, we must mention the work by Murray and Chu [2]. They investigated the drone-assisted parcel delivery problem, where customers can be served by either a driver-operated truck or an unmanned aircraft carried by the truck [2]. However, due to the load limitation of the unmanned aircraft, it can only visit one customer in each flight, which is not an exact two-echelon routing problem. Ha et al. [25,26] solve a routing problem facilitated by the adoption of an unmanned aircraft for last-mile delivery, where the UAV can also only serve one customer in a flight with the limitation of current carrying capacity. It is possible for the UAV to cover multiple check points in ISR missions. Another important contribution on the optimization for the cooperation of the GV and UAV is the work by Savuran and Karakaya, which investigated the routing problem for an UAV deployed on a GV to conduct ISR missions [9,30]. In their problem, it is possible for the UAV to serve multiple targets in one flight, but the GV’s route is fixed to the straight line road and the optimized decision is where should the UAV be launched and recycled.

From the above analysis, we can see that 2E-GU-RP is a new problem which is much more complex and difficult to solve than past problems. As far as we know, there are no literature studies of the proposed 2E-GU-RP. Motivated by the requirement in practical applications and the theoretical gap in the current literature, we study the 2E-GU-RP problem where the UAV conducts ISR missions and is capable of visiting multiple targets in each flight. To the best of our knowledge, our study is the first attempt to jointly optimize the cooperative routes for a GU and UAV, where the spatial and temporal synchronization are considered. 

## 3. Problem Formulation

The two-echelon cooperated ground vehicle and its carried unmanned aerial vehicle routing problem (2E-GU-RP) considers a set of targets, each of whom must be visited (or served) exactly once by the UAV. In our problem, all the targets are incapable of direct surveillance by GV, the progress of the whole ISR mission can be described as follows: The GV with the UAV starts from a main depot or a base and cruises a loop on the road. As the GV cruises, the UAV starts a flight by launching from the GV, then flies automatically to visit some targets, and finishes the ISR missions as planned, finally landing at the GV to change or charge its battery for the next flight. The 2E-GU-RP should model the cruising route of the GV, the assignment of each flight, and the UAV route in each flight. The mathematical model is formulated as follows.

The notations used in the model formulation are presented as follows:

**Notations:**GThe complete undirected graphVThe set of all nodes$V_{s}$The set of all rendezvous nodes$V_{t}$The set of all rendezvous nodesEThe set of all edges$E_{1}$The subset of edges that GV gets through$E_{2}$The subset of edges that UAV gets through$s_{j}$The service time of target j$t_{ij}$The travelling time between nodes *i* and *j*θThe maximal working time of the UAV

The 2E-GU-RP can be defined as a complete undirected graph G=(V,E). V is the set of all nodes including one main depot (node 0), a subset $V_{s}=\left\{1,2,\ldots ,m\right\}$ of *m* possible rendezvous nodes, and a subset $V_{t}=\left\{m+1,m+2,\ldots ,m+n\right\}$ of *n* customer nodes (targets). Each rendezvous node represents a location (generally, it is a parking lot) in the road network, where the GV can stop for recycling and launching the UAV. Each target $j\in V_{t}$ has a known service time $s_{j}$ for the UAV to complete the ISR mission on its target.

For each edge (i,j) where $i,j\in \left\{0\right\}\cup V_{s}$, let it denote a shortest path in the actual road network and the ground vehicle can only travel on these edges and consume a known travelling time $t_{ij}$. The UAVs can travel all of the edges in *E*, but it is not necessary for them to travel the edges in the road network. For convenience in the routing progress of the UAV, we only consider edge (i,j), where $i,j\in V_{t}$ or one of them belongs to $\left\{0\right\}\cup V_{s}$ but not both, and let (i,j) denote a shortest (usually linear) path between nodes *i* and *j*, which is also associated with a known travelling time $t_{ij}$. Thus, there are two kinds of edges in *E*, and we let $E_{1}$ be the subset of edges for the ground vehicle and $E_{2}$ be the subset of edges for UAVs, and also $E=E_{1}\cup E_{2}$.

**Figure 4:**
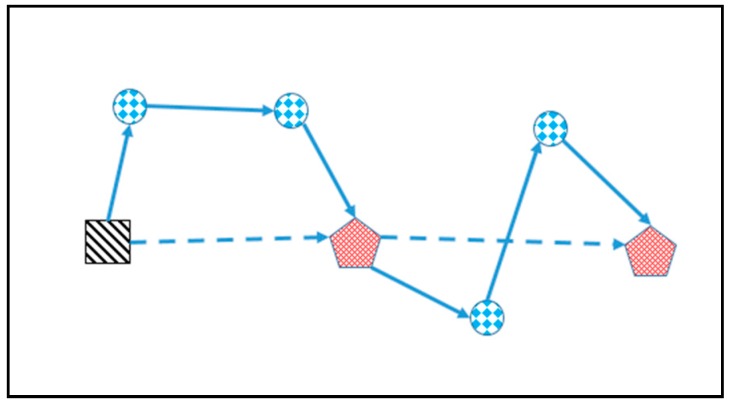
Schematic diagram of the discussed situation.

The rendezvous node is actually a small area which is wide enough for the GV recycling or launching the UAV where GV can stop and does not impact the traffic. Thus, there are usually sufficient rendezvous nodes in the road network, and we assume that the GV can always find a rendezvous node in the following trip for recycling the UAV after it launches the UAV from some node, which means that each road arc traversed by the GV, as depicted in Figure 4, corresponds to a flight route of the UAV. The distribution of rendezvous nodes is quite dense in our problem, and thus for most of the situation this assumption is acceptable.

The following assumptions are introduced to develop the model for 2E-GURP:

- The positions and service times for all targets are known.
- All targets must be visited and each target can only be visited once.
- There is an endurance restriction for UAV, which is known, and there is no endurance limitation for GV.
- The GV can only traverse arcs in the road network.
- When UAV returns to GV, UAV can change its battery and start next flight immediately. The time of changing battery is very short and negligible.
- Each road arc traversed by the GV corresponds to a flight route of the UAV.

In order to formulate the 2E-GU-RP problem, we define the following variables:

$x_{ij}$: binary variable $i,j\in \left\{0\right\}\cup V_{s}$ that is equal to 1 if edge $\left(i,j\right)\in E_{1}$ is traversed by the ground vehicle;

$y_{ij}$: binary variable i,j∈V that is equal to 1 if edge $\left(i,j\right)\in E_{2}$ is traversed by the UAV.

$z_{ijk}$: binary variable $i\in V_{t},j,k\in \left\{0\right\}\cup V_{s}$ that is equal to 1 if target *i* is visited by the UAV in the flight which starts from rendezvous node *j* and ends at rendezvous node *k*.

The 2E-GURP can be formulated as the following 0–1 integer programming in Model 1:
**Model 1.** Integer ProgrammingMinimize: $\sum_{i\in V}\sum_{j\in V}\left(y_{ij}\times t_{ij}\right)$(0)Subject to:$\sum_{i\in \left\{0\right\}\cup V_{s}}x_{ij}=\sum_{i\in \left\{0\right\}\cup V_{s}}\;x_{ji}\leq 1,\;\forall j\in \left\{0\right\}\cup V_{s},$(1)$\sum_{i\in V_{s}}x_{i0}=\sum_{i\in V_{s}}x_{0i}=1,$(2)$\sum_{i\in S}\sum_{j\in S}x_{ij}<\left|S\right|,\;\forall S\subset \left\{0\right\}\cup V_{s}$(3)$z_{ijk}\leq x_{jk},\;\forall i\in V_{t},\forall j,k\in \left\{0\right\}\cup V_{s,}$(4)$\sum_{j\in \left\{0\right\}\cup V_{s}}\sum_{k\in \left\{0\right\}\cup V_{s}}z_{ijk}=1,\;\forall i\in V_{t},$(5)$\sum_{i\in V}y_{ij}=\sum_{i\in V}y_{ji}=1,\;\forall j\in V_{t,}$(6)$\sum_{i\in V}y_{ij}=\sum_{i\in V}y_{ji}\leq 1,\;\forall j\in \left\{0\right\}\cup^{\text{​}}V_{s},$(7)$\sum_{i\in \left\{0\right\}\cup V_{s}}\sum_{j\in V_{t}}y_{ij}=\sum_{i\in \left\{0\right\}\cup V_{s}}\sum_{j\in V_{t}}y_{ji}=\sum_{i\in \left\{0\right\}\cup V_{s}}\sum_{j\in \left\{0\right\}\cup V_{s}}x_{ij},$(8)$\sum_{j\in V_{t}}y_{ij}=\sum_{j\in \left\{0\right\}\cup V_{s}}x_{ij},\;\forall i\in \left\{0\right\}\cup V_{s},$(9)$\sum_{k\in \left\{0\right\}\cup V_{s}}z_{ijk}\geq y_{ji},\forall i\in V_{t},\;\forall j\in \left\{0\right\}\cup V_{s},$(10)$\sum_{j\in \left\{0\right\}\cup V_{s}}z_{ijk}\geq y_{ik},\forall i\in V_{t},\;\forall k\in \left\{0\right\}\cup V_{s},$(11)$2\times y_{ij}\leq \max\left\{z_{iab}+z_{jab}\right\},\;\forall i,j\in V_{t},\;\forall a,b\in \left\{0\right\}\cup V_{s}$, (12)$\sum_{j\in S_{1}}\sum_{j\in S_{1}}y_{ij}<\left|S_{1}\right|,\;\forall S_{1}\subset V,$(13)$t_{ab}\times x_{ab}\leq \sum_{i}\left(z_{iab}\times s_{i}\right)+\sum_{i\in R\cup \left\{a,b\right\}}\sum_{j\in R\cup \left\{a,b\right\}}\left(y_{ij}\times t_{ij}\right)\leq \theta ,\;\forall a,b\in \left\{0\right\}\cup V_{s},\;R=f\left(a,b\right),$(14)$x_{ii}=0,\;\forall i\in \left\{0\right\}\cup V_{s},$(15)$y_{ii}=0,\;\forall i\in V,$(16)$y_{ij}=0,\;\forall i,j\in \left\{0\right\}\cup V_{s},$(17)$x_{ij},\;y_{ij},\;z_{ijk}\in \left\{0,1\right\},\;\forall i\;\in V_{t},\;j,k\in \left\{0\right\}\cup V_{s}.$


Objective function (0) minimizes the total routing time for the UAV. Constraints (1)–(3) restrict the GV’s routing progress. Constraint (1) ensures the connectivity for the ground vehicle in the first-level trip. We limit that each rendezvous depot can be visited no more than once to guarantee the readability of final answer. Constraint (2) illustrates that the GV must depart and end at the main depot. Constraint (3) ensures that every subset of rendezvous nodes (noted as *S*) is incapable of having a closed loop. 

Constraints (4) and (5) impose the assignment limit on each rendezvous node, target *i* can only be assigned to the edge *(j*, *k)* where the GV cruised. Every target point is assigned to a flight only once. Sets of Constraints (6) and (7) state that every target point is visited only once and only a part of the rendezvous nodes are also visited for taking off and landing. In the situation we discussed above, Constraint (8) guarantees that each flight launches and takes off only once. Constraint (9) states that second-level trips start from the point of the GV route. Sets of Constraints (10) and (11) reinforce Constraints (4) and (5) that the launching or landing progress performs only when the target is assigned to the edge *(j*, *k)*. Constraint (12) ensures the UAV route from target *i* to *j* occurs only when they are in the same flight, which means if the UAV flies from target *i* to *j*, there is a flight that starts from rendezvous node a and ends at rendezvous node *b*, and both *i* and *j* are assigned to this flight (in this condition, $\max\left\{z_{iab}+z_{jab}\right\}=2$, otherwise $\max\left\{z_{iab}+z_{jab}\right\}=1$). Constraint (13) prevents subtours in second-level trips. Every subset of target nodes (here noted as $S_{1}$) is incapable of having a closed loop.

For any edge $\left(a,b\right)\in E_{1}$, let f(a,b) denote a subset of target nodes. If $z_{iab}=1$, then target node *i* belongs to f(a,b). Since we restrict that every target node is assigned to an edge only once in the Constraint (5), the set $V^{\prime }=\left\{f\left(a,b\right)\right\},\;\;\forall a,b\in \left\{0\right\}\cup V_{s}$ divides $V_{t}$ into plenty of subsets. Constraint (14) guarantees that when the UAV finishes its mission the GV is ready to pick up the UAV at the appointed place. Meanwhile, there is a battery limit for the UAV in each flight. The maximal working time of the UAV is noted as θ. Sets of Constraints (15) and (16) impose that it is a simple graph, and Constraint (17) guarantees the UAV does not fly directly between rendezvous nodes.

The objective directly minimizes the total routing time for UAV. However, Constraints (4), (8) and (9) together ensure that each flight of UAV corresponds to one traversed arc of GV, and Constraint (14) ensure that the GV’s traversing time for that arc is no more than the total time of the corresponding flight of UAV. Thus, the objective indirectly minimizes the routing time for GV, and makes sure the GV traverses the shortest route that can cooperate with the flights of UAV.

## 4. Heuristics

A current survey of two-echelon location routing problems shows that most problems are solved by heuristic algorithms and exact algorithms can only be used to solve small size problems where the number of depots used for stopping is usually less than 5 [18]. Also, exact algorithms require very long computational time for solving small-size instances, which is not acceptable for our application. Thus, we propose two constructive heuristic algorithms for the 2E-GU-RP. The first heuristic (H1) algorithm starts by constructing a complete tour for all of the targets in the second echelon, and then applies a splitting procedure to cut this route into a set of subtours which can cooperate with the routing of the GV. The genetic algorithm proposed by Barrie and Ayechew [31] for solving the VRP is utilized to build a tour for all of the targets. The second heuristic (H2) algorithm starts by constructing a complete tour for all of the rendezvous nodes in the first echelon, and then assigns all of the targets to the edges in this tour subject to the endurance of the UAV. Similarly, a genetic algorithm is utilized to build the tour for rendezvous nodes. More detail of H1 and H2 are presented in the following subsections.

### 4.1. H1: Construct Target Visiting Tour and Split

**Figure 5:**
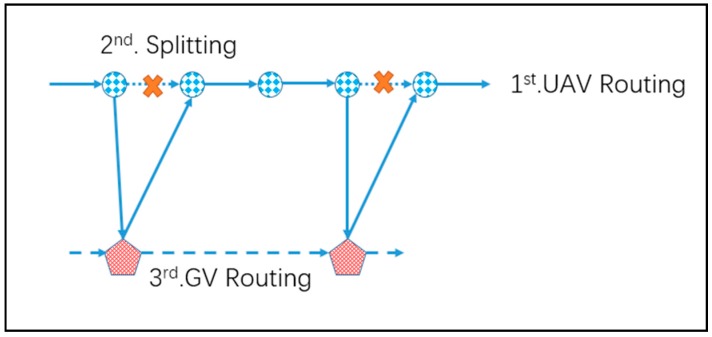
Illustration of the searching process for H1.

If we solve the TSP for all the targets and obtain the short time for visiting all targets in one route, it is obvious that this time forms a low bound of the 2E-GU-RP, which means we cannot use less time to finish visiting all of the targets. Based on this observation, a commonly used idea for solving TSP-related problems is to find a complete route for all targets and then “split” it into subtours that can satisfy the constraints on the problem [32]. For more details of the idea and procedure, readers are suggested to refer to the review on route-first split-second heuristic approaches presented by Prins [33]. The basic idea for H1 is also similar, which is presented in Figure 5.

We first construct a complete tour, *T*, for visiting the initial depot {0} and all of the targets $V_{t}$ without considerations of the endurance of the UAV. In this tour, we make sure the Constraints (6), (7) and (13) were held. Secondly, we search the tour *T* in a sequence and structure a subset of the visited target nodes. We create an indicator variable to count the time of the tour and service. If the indicator variable exceeds the restriction of the UAV’s endurance in Constraint (14), we search a set of available rendezvous nodes for the UAV to charge its battery and ensure that the UAV is capable of landing on the GV in those nodes. We treat those rendezvous nodes as a layer of leaf nodes. Then we find the nearest and unmarked rendezvous node among the leaf nodes, reset the indicator variable, and restart the search from this node. If there is no leaf node available, we could delete the last target node. If there is no target node remaining in this layer, we could remount to the upper layer (parent nodes) and mark this node. We repeat the above loop until an available solution is found. Finally, we build the tour of the selected rendezvous node under the Constraints (1)–(4), and check the rest of constraints.

To implement our algorithm, this tree structure has four members: a pointer to the parent node, the number of this node, a variable P to indicate the serial number of the giant tour, and a list of leaf nodes. Moreover, we need a list to mark the target node that is visited, using the pointer *nt* to indicate the tree node at present. The main procedure for H1 is as follows:

**Heuristic 1 (H1)**:1 DO 2 Subset=F(nt,T,P);// build a subset for *nt* 3   P=P+|Subset|; 4     DO 5      nt.leaf=findleaf(list,Subset);//find the set of leaf nodes 6      IF Subset==∅ 7        mark(nt);nt=nt.parent;P=nt.P; 8        BREAK; 9      END IF 10      J=find(nt.leaf,T,P);//find the nearest node 11      IF J==∅ 12        Delete(Subset);P=P−1; 13        CONTINUE; 14      END IF 15     WHILE J~=∅ 16     IF Subset==∅ 17       CONTINUE; 18     END IF 19     nt={nt.num,J,P,∅};list(J)=1; 20 WHILE P == n + 1

### 4.2. H2: Construct Rendezvous Node Tour and Assign

**Figure 6:**
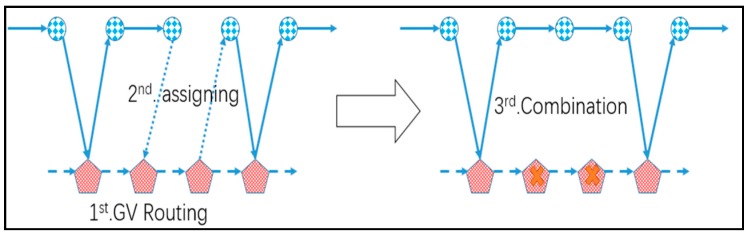
Illustration of the searching process for H2.

In H2, we first construct a complete tour covering all rendezvous nodes in the road network to ensure that Constraints (1)–(3) are verified. Then assign the targets to the arcs traversed by the GV subject to the endurance of the UAV under the Constraints (4), (5), (10) and (11). Finally, we combined the adjacent edge of the vehicle tour and built the UAV tour under the Constraints (6)–(9), (13). The basic idea of H2 is illustrated in Figure 6.

The basic idea of H1 comes from the algorithms proposed in [34,35], which are utilized to solve the location and routing problem. Firstly, we built a tour *T* to cover the initial depot {0} and all rendezvous nodes $V_{s}$. For each edge included in *T*, we structure a set to indicate the assignment and use Set (i) to signify the *i*th set. Secondly, we assign all targets to the nearest rendezvous node set by the function Findmin(T, I) to find a set satisfying the UAV’s endurance and having the shortest distance to the target I and the function Add (Set (J), I) to add target I into Set (J). Finally, we combine the adjacent edge of the vehicle tour judged by the function t (Set) to count the time of the tour and service of the set and make sure the Constraint (14) is verified. The main procedure for H2 is as follows.

**Heuristic 2 (H2):**1 I=m+1 2 DO 3   J=Findmin(T, I) 4   Add(Set(J), I) 5   I=I+1 6 WHILE I>m+n 7 I=1 8 DO 9   IF $t\left(\mathrm{Set}\left(I\right)\cup^{\text{​}}\mathrm{Set}\left(I+1\right)\right)<\theta$ 10     Combine(Set(I),Set(I+1)) 11   END IF 12   I=I+1 13 WHILE I>m

## 5. Computational Study

In this section, a number of instances with different sizes are randomly generated to test the proposed two algorithms, and the computational results are compared and analyzed.

### 5.1. Experiment Design

The programs for both algorithms are coded in MATLAB R2015a 8.5.0 with a personal license. All of the computational experiments are conducted on a Dell laptop with an Intel 4 processor clocked at 2.6 GHz, 4 GB of memory, and Windows 7 OS. As far as we know, no public instance is available for the 2E-GU-RP, and we randomly generated two sets of Euclidean instances with different sizes to test the proposed algorithms, in which the random generation methods are commonly utilized in the literature on VRP [10].

In the first set, the positions of all rendezvous nodes and targets are randomly generated in a two-dimensional square space. There are six different sizes for the test instances, as shown in Table 1, and we randomly generated 100 instances in each scale. The service time of each target is uniformly distributed between 5 and 20 time units. The UAV has 100 units of maximal working time with the fixed speed of 1-unit distance per unit time, while the GV travels with a fixed speed of 1-unit distance per unit time. There are arcs between any two rendezvous nodes (targets), and the travelling time is calculated based on the distance and the velocity of the GV (UAV).

In the second set, the problem sizes and the value of parameters generated are the same with the first set, and the only difference is the generation method for the positions of nodes. We equally divide the square space into four square subfields, and randomly generate the rendezvous nodes separately in each subfield with an equal scale of nodes, which can make the distribution of the nodes more dispersed then that in the first set.

Both H1 and H2 involve a progress of constructing a tour, which could be simply solved by using an existed algorithm to solve a TSP. To implement our heuristics, we apply a genetic algorithm (GA) setting the population size at 200 and iterating 1000 generation, by which we assume the GA will give at least a locally optimal solution.

### 5.2. Comparison with the Exhaustive Method

#### 5.2.1. Exhaustive Method

To solve this problem optimally, we design an exact algorithm and compare its efficiency and effectiveness with the proposed algorithms. Initially, we analyze the constraints in the model and their impacts on the decision variables. Note P as the number of permutations and C as the combinatorial number. Firstly, restricted by the Constraint (6), the number of possible routes for visiting all targets is $P_{n}^{n}$. For each of these routes, there are at least 1 depot and at most m depots in each stopping strategy, considering Constraint (1). The number of stopping node selection strategies is $\sum_{i}^{m}C_{m}^{i}$. For each stopping selection strategy including *i* stopping nodes, the number of possible cooperation strategies for GV and UAV is $C_{n-1}^{i}$, and the number of routing strategies for GV visiting these *i* nodes is $P_{i}^{i}$. Thus, the total number of possible solutions is $P_{n}^{n}\times \sum_{i}^{m}\left(C_{m}^{i}\times C_{n-1}^{i}\times P_{i}^{i}\right)$. For each possible solution, we should check whether it satisfies all the constraints and find the feasible solution. Finally, all feasible solutions are compared to obtain the optimal one. 

According to the above analysis, we construct a tree structure for searching all the feasible solutions. The first level of leaf node is constructed by the permutations of all targets. Each leaf node represents one of the permutations. We start our search progress from the leaf nodes in the second level: we search all the selectable positions in order and insert a depot. This depot separates the UAV route into two parts (flights). If both the former flight and the later one satisfy the constraints, we find a feasible solution. If the former one violates the constraints, we select another depot as the substitution and recheck. If the later one violates the constraints, we iterate the search progress by searching the next selectable position. To increase the efficiency of this depth-first search program, we add some pruning rules, such as using an array to sign the visited depots and exclude them from the next searching.

#### 5.2.2. Experiment Design and Computational Results

Table 2 presents some small-size instances and their solution space analysis. When we utilized the exhaustive method to solve instances for 12 stopping nodes and 25 targets, we found it cannot obtain the optimal solution in several hours. Thus, we only randomly generated 10 instances for each small-size problem with no more than 8 targets which were detailed in the following Section 5.3.2. All these small instances are solved by the exhaustive method and our heuristics, and the results are presented in Table 9, where the best values in each instance are shown in bold.

From Table 3, we can see that the computational time of the exhaustive method is very long, and for most of the instances with 8 targets the time taken is over 2 h. For all 30 instances, H1 obtains the optimal solutions for 18 instances while H2 finds the optimal solutions for 17 instances. For most other instances, both H1 and H2 obtain nearly optimal solutions with quite a small gap between them and the optimal solution presented by the exhaustive method. However, the computational time for H1 and H2 is much less than that of the exhaustive method. Thus, H1 and H2 is more suitable for practical applications.

### 5.3. Computational Results and Analysis

#### 5.3.1. Dataset 1

All 600 instances in Dataset 1 are solved by H1 and H2. To compare the overall performance of H1 and H2, we run 100 times to solve each of the instances. In each run we, respectively, record the objective value obtained by H1 and H2 and the CPU time they consume. Firstly, we randomly select one instance in each size and analyze the results for all 100 runs to compare their veracity and stability. Then we check the solving progress among all instances to decrease the impact of the randomness in data generation. 

We firstly compare the solutions obtained by H1 and H2 for all 100 runs. Table 4 summarizes the performance on the solutions obtained of H1 and H2, e.g., for the first instance in the table, H1 obtains a better solution than that of H2 in 56 runs. In Table 1, it can be seen that for instances 2 and 5, H2 performs better, while H1 performs better for the other four instances. 

**Figure 7:**
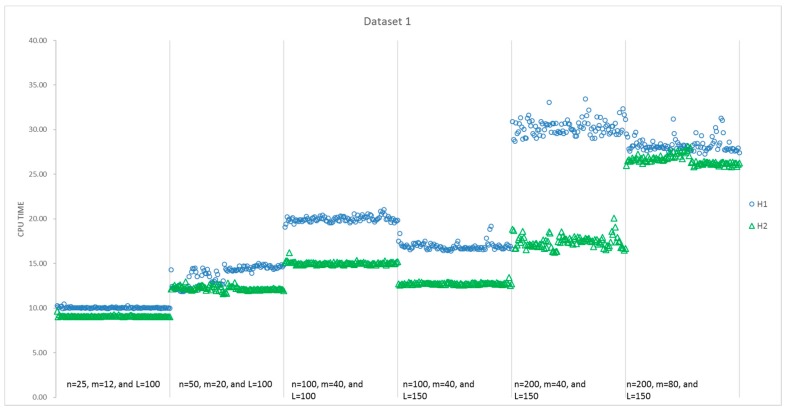
Comparison of CPU Time for H1 and H2 in the first dataset.

**Figure 8:**
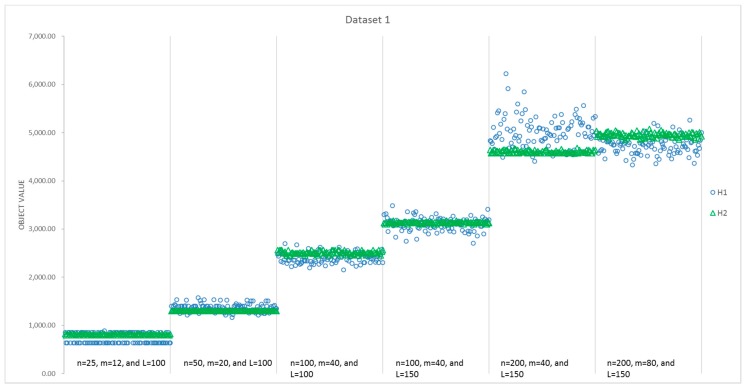
Comparison of objective values for H1 and H2 in the first dataset.

The results of all 100 runs for each instance size obtained by H1 and H2 are summarized in Table 5. Figure 7 presents a detailed comparison of the computational time consumed by H1 and H2 for instances with different sizes, while Figure 8 presents a comparison of the obtained value of the objectives. It can be seen that the average CPU time of H2 is less than that of H1 for the first five instances with different sizes, and all of the standard deviations of the 100 runs for H2 are smaller than that of H1. However, the average objective value obtained by H1 is better than that of H2, except the second and fifth instances. Moreover, the best solutions are always found by H1 in Figure 8, that is, H1 can present more optimal solutions than H2 for most of the instances. Additionally, 57% of the solutions for all six instances indicate that H1 performs better in Table 4. However, things are different with respect to stability. H2 performs more stably, as the standard deviation is much less than H1 in every instance. With respect to the average, H2 performs better in the second and fifth instances. Thus, for these six instances, H1 is able to find better objectives with a higher computational time.

To eliminate the impact of randomness in data generation, we test H1 and H2 in all 600 instances. Every instance is solved 100 times by both H1 and H2, respectively, and we select the optimal answer and its CPU time of each algorithm as the indictors for their performance. To summarize the performance, we calculate the average and standard deviation among 100 instances in each size, and the results are presented in Table 6. There are few differences between H1 and H2, e.g., for the fifth instance size in Table 6, their differences in the average objective values are less than 2%. Generally speaking, H1 is capable of finding a slightly better solution, but H1 is not a stable algorithm and consumes more time.

#### 5.3.2. Dataset 2

H1 and H2 are also utilized to solve the randomly-selected instance for each size in the second dataset and, for each instance, H1 and H2 run 100 times, respectively, in the same way. The results on CPU time and the objective values are summarized in Table 7. In Dataset 2, H1 performs much better. For all six instances in the Table 8, H1 obtains better solution than that of H2 in more than 50 runs.

**Figure 9:**
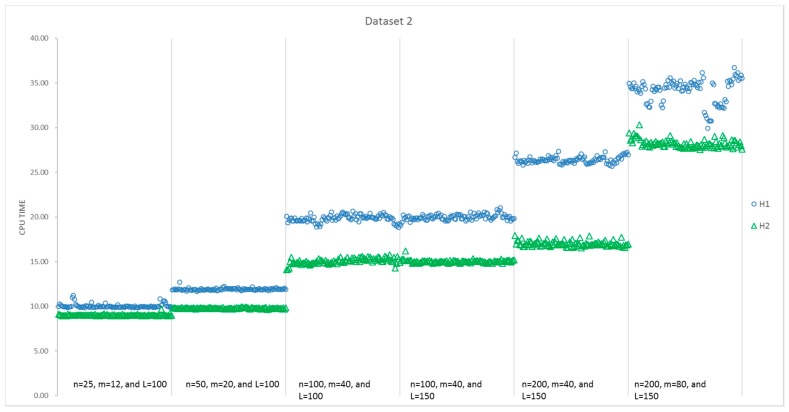
Comparison of CPU time for H1 and H2 in the second dataset.

**Figure 10:**
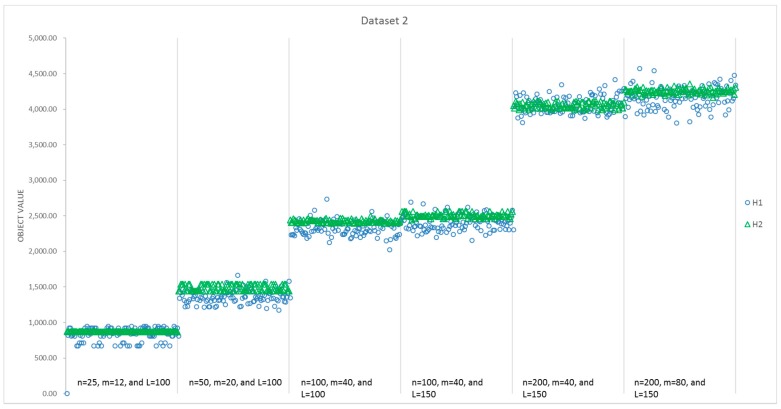
Comparison of objective value for H1 and H2 in the second dataset.

The results of all 100 runs obtained by H1 and H2 are summarized in Table 6. Figure 9 presents a detailed comparison of the computational time consumed by H1 and H2 for instances with different sizes, while Figure 10 presents a comparison on the obtained objective values. It can be seen that the average CPU time of H2 is less than that of H1 for all six instances with different sizes, and all of the standard deviations of the 100 runs for H2 are smaller than that of H1. We can draw a similar conclusion that H1 obtains a capability to find better objectives with a higher computational time. Additionally, the objective values and CPU time in Dataset 2 cost more than those in Dataset 1.

To decrease the impact of randomness of data generation, we also test H1 and H2 in all 600 instances of Dataset 2, and the summary is shown in Table 9. Dataset 2 ensures that the randomized generated data would not be centralized, thus, the objective values are more than those in H1. However, the division increases the objective value and its standard deviation. There are many more difference between H1 and H2, e.g., for all six instance sizes in Table 9, there is at least a 10% difference in the average objective value. Generally speaking, H1 is capable of finding a better solution, but H1 is not a stable algorithm and consumes more time.

## 6. Conclusions

UAVs have been widely used for military and civilian purposes, such as surveillance, bound patrolling, search and rescue in the wilderness, and forest fire prevention or reconnaissance. When a GV cooperatively works with a UAV and acts as a moving depot that provides service for launching, recycling, and changing batteries, it greatly increases the capability and efficiency of UAVs for completing tasks in these areas. In this paper, we studied a two-echelon routing problem for the ground vehicle and its unmanned aerial vehicle. A 0–1 integer programming model is developed to formulate the problem, where the routing constraints and the interaction between the two-echelon routes are considered. This model is extensible and can be applied to other cooperated routing problem. Two heuristics are proposed to solve the model, whose performances are compared through random instances with different scales. Experimental results show that H1 can obtain better solutions than H2 most of the time, while its computational time is longer than that of H2. To extend the computational study, we construct 30 small-scale instances and compare the heuristics with an exhaustive method. Experimental results show that both heuristics are effective and efficient.

To extend the proposed study in this paper, future directions of research could consider the 2E-GU-RP with time windows and develop other heuristics. In many practical applications, there are time window constraints on visiting the targets, which would further limit the routing process of the GV and UAV. This would be a meaningful extension to the development of a model and solution algorithm for this problem with a time window or in a dynamic situation. In model formulation, we introduced six assumptions. It would be a meaningful extension to modify the model by discarding or changing the assumptions in practical applications. In this paper, we develop two heuristics for 2E-GU-RP that can help find better, feasible solutions. In future research, studying new algorithms, especially the algorithms that can obtain tight lower bounds, such as Lagrangian heuristics and branch and cut algorithms, would be quite useful, and it would be a promising development of current work to investigate efficient solution approaches for solving the model in a multiple-UAVs situation.

## Figures and Tables

**Table 1:** The sizes for the tested instances.

Size No.	Number of Targets (n)	Number of Rendezvous Nodes (m)	Length of Square (L)
1	25	12	100
2	50	20	100
3	100	40	100
4	100	40	150
5	200	40	150
6	200	80	150

**Table 2:** Size of the instances and their solution space analysis.

Size Number	Stopping Node	Target	Solution Space
1	3	6	97,200
2	3	7	1,149,120
3	3	8	14,394,240
Instance size 1	12	25	3.25 × 10^39^

**Table 3:** Comparison of the results for the exhaustive method and our heuristics.

Size	Instance	Exhaustive Method		H1		H2	
		CPU Time	Value	CPU Time	Value	CPU Time	Value
**1**	**1**	61.85	**321.89**	**1.51**	**321.89**	1.58	**321.89**
	**2**	79.14	**300.90**	**1.50**	**300.90**	1.61	309.34
	**3**	43.35	**389.26**	1.51	**389.26**	**1.51**	**389.26**
	**4**	51.14	**312.03**	**1.53**	348.59	1.56	**312.03**
	**5**	59.56	**337.31**	1.58	**337.31**	**1.48**	**337.31**
	**6**	57.99	**406.57**	1.48	**406.57**	**1.48**	**406.57**
	**7**	66.35	**290.38**	**1.54**	317.36	1.62	**290.38**
	**8**	91.85	**317.31**	**1.50**	**317.31**	1.54	336.07
	**9**	48.73	**370.72**	**1.54**	**370.71**	1.56	**370.71**
	**10**	48.34	**324.18**	**1.51**	366.93	1.59	365.36
**2**	**1**	815.01	**272.12**	**1.47**	**272.12**	1.48	292.50
	**2**	675.69	**336.45**	1.39	**336.45**	**1.36**	**336.45**
	**3**	915.01	**295.36**	1.39	**295.36**	**1.39**	297.48
	**4**	658.59	**355.45**	1.33	401.03	1.45	**355.45**
	**5**	547.25	**343.35**	1.40	444.85	**1.37**	**343.35**
	**6**	633.01	**322.97**	1.40	**322.97**	**1.39**	346.98
	**7**	777.82	**268.87**	**1.39**	321.86	1.40	309.96
	**8**	588.39	**344.39**	**1.33**	**344.39**	**1.33**	358.31
	**9**	611.49	**387.75**	1.37	**387.75**	**1.36**	421.18
	**10**	511.79	**394.88**	1.43	**394.88**	**1.40**	410.25
**3**	**1**	9791.31	**312.49**	1.58	334.58	**1.48**	342.36
	**2**	8813.26	**334.60**	1.54	364.40	**1.50**	**334.60**
	**3**	10562.73	**312.49**	1.79	334.58	**1.65**	342.36
	**4**	11922.39	**286.50**	1.45	**286.50**	**1.45**	**286.50**
	**5**	7850.43	**337.04**	1.42	370.34	**1.39**	**337.04**
	**6**	7296.56	**386.97**	**1.45**	428.58	1.47	**386.97**
	**7**	8447.61	**395.55**	1.44	402.41	**1.44**	435.12
	**8**	8338.28	**420.42**	**2.37**	**420.42**	2.48	**420.42**
	**9**	5438.58	**388.81**	1.67	**388.81**	**1.47**	**388.81**
	**10**	8640.46	**299.94**	1.63	**299.94**	**1.61**	**299.94**

**Table 4:** Percentage of solutions obtained by H1 which are better than that of H2.

Instance Size	1	2	3	4	5	6	Average
H1 better	56%	20%	84%	53%	8%	91%	52%

**Table 5:** Computational results for instances in Dataset 1.

Instance Size	CPU Time				Objective Value			
	Average		Standard Deviation		Average		Standard Deviation	
	H1	H2	H1	H2	H1	H2	H1	H2
**1**	10.05	9.08	0.08	0.07	734.11	809.98	110.11	11.66
**2**	13.83	12.22	0.98	0.24	1372.69	1298.55	85.79	0
**3**	20.00	15.04	0.32	0.17	2397.61	2505.56	106.88	31.90
**4**	16.92	12.74	0.43	0.11	3104.42	3131.83	140.89	16.50
**5**	30.28	17.43	0.87	0.64	5037.44	4602.18	312.16	26.75
**6**	28.29	26.60	0.77	0.50	4730.40	4954.15	179.10	47.23

**Table 6:** Results of all instances in Dataset 1.

Instance Size	CPU Time				Objective Value			
	Average		Standard Deviation		Average		Standard Deviation	
	H1	H2	H1	H2	H1	H2	H1	H2
**1**	10.34	9.42	0.18	0.09	743.87	887.52	81.47	61.34
**2**	13.71	11.25	1.03	0.81	1275.20	1462.06	81.40	79.11
**3**	17.59	13.37	0.35	0.23	2191.49	2385.94	85.63	50.10
**4**	22.41	19.06	6.15	6.65	3991.13	4058.99	114.03	97.95
**5**	28.99	25.86	0.57	0.49	4999.91	5221.51	196.96	85.54
**6**	37.20	31.03	12.60	9.23	3943.52	4116.25	102.1	72.83

**Table 7:** Results for the computational study of Dataset 2.

Instance Size	CPU Time				Objective Value			
	Average		Standard Deviation		Average		Standard Deviation	
	H1	H2	H1	H2	H1	H2	H1	H2
**1**	10.03	9.01	0.24	0.09	831.17	870.94	93.80	2.52
**2**	11.92	9.80	0.12	0.55	1354.00	1482.84	92.03	45.04
**3**	19.84	15.08	0.39	0.32	2321.09	2420.91	106.36	20.89
**4**	20.00	15.04	0.32	0.17	2397.61	2505.56	106.88	31.91
**5**	26.40	17.01	0.36	0.29	4062.53	4051.29	124.21	42.10
**6**	34.14	28.22	1.37	0.46	4161.45	4253.42	150.74	33.36

**Table 8:** Percentage that H1 is better than H2 in objective value.

Instance Size	1	2	3	4	5	6	Sum
H1 better	61%	93%	86%	84%	53%	77%	76%

**Table 9:** Results for computational study of Dataset 2.

Instance Size	CPU Time				Objective Value			
	Average		Standard Deviation		Average		Standard Deviation	
	H1	H2	H1	H2	H1	H2	H1	H2
**1**	12.48	11.42	0.11	0.12	785.7	873.7	68.27	48.54
**2**	14.60	12.36	0.09	0.09	1283.4	1417.5	74.87	60.56
**3**	19.79	16.26	0.15	0.15	2168.2	2354.4	75.86	55.76
**4**	16.52	13.48	0.48	0.43	2410.7	2940.1	108.26	63.96
**5**	27.10	16.41	0.92	0.68	4435.8	4968.4	124.71	89.35
**6**	28.15	27.35	0.82	1.81	4300.4	4815.5	106.36	73.40
